# An efficient heuristic method for active feature acquisition and its application to protein-protein interaction prediction

**DOI:** 10.1186/1753-6561-6-S7-S2

**Published:** 2012-11-13

**Authors:** Mohamed Thahir, Tarun Sharma, Madhavi K Ganapathiraju

**Affiliations:** 1 Department of Biomedical Informatics, School of Medicine, University of Pittsburgh, Pittsburgh, PA, USA; 2 Intelligent Systems Program, School of Arts and Sciences, University of Pittsburgh, Pittsburgh, PA, USA; 3 Language Technologies Institute, Carnegie Mellon University, Pittsburgh, PA, USA

## Abstract

**Background:**

Machine learning approaches for classification learn the pattern of the feature space of different classes, or learn a boundary that separates the feature space into different classes. The features of the data instances are usually available, and it is only the class-labels of the instances that are unavailable. For example, to classify text documents into different topic categories, the words in the documents are features and they are readily available, whereas the topic is what is predicted. However, in some domains obtaining features may be resource-intensive because of which not all features may be available. An example is that of protein-protein interaction prediction, where not only are the labels ('interacting' or 'non-interacting') unavailable, but so are some of the features. It may be possible to obtain at least some of the missing features by carrying out a few experiments as permitted by the available resources. If only a few experiments can be carried out to acquire missing features, which proteins should be studied and which features of those proteins should be determined? From the perspective of machine learning for PPI prediction, it would be desirable that those features be acquired which when used in training the classifier, the accuracy of the classifier is improved the most. That is, the *utility *of the feature-acquisition is measured in terms of how much acquired features contribute to improving the accuracy of the classifier. Active feature acquisition (AFA) is a strategy to preselect such instance-feature combinations (i.e. protein and experiment combinations) for maximum utility. The goal of AFA is th*e creation of optimal training set *that would result in the best classifier, and not in determining the best classification model itself.

**Results:**

We present a heuristic method for active feature acquisition to calculate the utility of acquiring a missing feature. This heuristic takes into account the change in belief of the classification model induced by the acquisition of the feature under consideration. As compared to random selection of proteins on which the experiments are performed and the type of experiment that is performed, the heuristic method reduces the number of experiments to as few as 40%. Most notable characteristic of this method is that it does not require re-training of the classification model on every possible combination of instance, feature and feature-value tuples. For this reason, our method is far less computationally expensive as compared with previous AFA strategies.

**Conclusions:**

The results show that our heuristic method for AFA creates an optimal training set with far less features acquired as compared to random acquisition. This shows the value of active feature acquisition to aid in protein-protein interaction prediction where feature acquisition is costly. Compared to previous methods, the proposed method reduces computational cost while also achieving a better F-score. The proposed method is valuable as it presents a direction to AFA with a far lesser computational expense by removing the need for the first time, of training a classifier for every combination of instance, feature and feature-value tuples which would be impractical for several domains.

## Background

Constructing a complete human protein-protein interaction (PPI) network (the '*interactome'*) can accelerate discovery in biomedical sciences and is crucial to the study of disease mechanisms and drug discovery. For example, proteins (genes) which are associated with a disease interact with other disease-related genes more closely in the interactome [1]; for this reason, protein-disease associations can be determined based on the network topological features such as the degree of a node (i.e. protein), average distance of the node from disease-related proteins etc. [2]. Several network-based approaches have been devised to determine gene-disease associations and functional modules using the interactome, including neighborhood based approaches, clustering/graph partitioning based methods and random-walks [3-6]. However, only a fraction of the whole human interactome is known today, calling for methods to discover hitherto-unknown PPIs [7,8].

Determining PPIs by high-resolution experimental methods is very resource intensive. High throughput methods such as yeast 2-hybrid and mass spectrometry methods have low assay-sensitivity (i.e. the interactions that they *can *detect is only a subset of all PPIs that exist) and even among those that they can, each screen identifies a further smaller subset of PPIs [9]. Computational methods are therefore necessary to complement the high-throughput methods to reconstruct the interactome expeditiously. Several computational systems have been developed for prediction of protein-protein interactions, particularly for yeast and human, using machine learning approaches [10-14]. These approaches employ statistical machine learning methods to classify whether two proteins interact with each other or not, based on the biological features of proteins such as their localization, molecular function and the tissues the proteins are expressed in. In all of these methods, it is assumed that a training data set is available, and that the pending goal is to develop an algorithm to learn to model the relation between feature space and labels given represented by the training data.

However, in the current training data many features are unknown (i.e. 'missing') for many proteins. Carrying out wet-lab experiments to determine all such missing features is infeasible as those experiments require human expertise, time, high-end equipment and other resources. It may however be possible to carry out a few experiments to determine some of the missing features, if not all. If only a few missing values can be determined, which features for which proteins should be determined by experiments? From the perspective of machine learning for PPI prediction, it would be desirable that those experiments be carried out which when used in training the classifier, the accuracy of the classifier is improved the most. That is, the *utility *of the feature-acquisition is measured in terms of how much acquired features contribute to improving the accuracy of the classifier. Active feature acquisition (AFA) is a strategy to preselect such instance-feature combinations (i.e. protein and experiment combinations) for maximum utility. It is to be noted that the goal of AFA is th*e creation of optimal training set *that would result in the best classifier, and not the determination of the best classification model itself. Subsequent to creation of training data with active feature acquisition, any state-of-the-art method such as random forest based methods may be applied to learn the classification model. While PPI prediction itself is being actively studied recently [11,12,15], AFA strategy has not been applied in this domain.

A few algorithms have been developed for AFA in other application domains which calculate utility of feature-acquisition based on the accuracy of the current model and its confidence in the prediction. Melville et al. proposed a framework for performing active feature acquisition [16], which is described here briefly. Here, the training set T of m instances is represented by the matrix F, where Fi,j corresponds to the value of the j-th feature of the i-th instance. The feature matrix initially has missing values, the class label of each instance is already known. Missing features may be acquired with active feature acquisition procedure at a cost of Ci,j for feature Fi,j. qi,j refers to the query for value of Fi,j [16]. The objective of AFA is to query for missing feature values such that the most accurate classifier is built for a given budget for feature acquisition. The framework proposed by Melville et.al [16], is an iterative model wherein in each iteration a set of missing features, which provide the highest expected improvement to classifier accuracy at minimal cost, are chosen and queried. Known feature values are added to training data and the classifier is retrained. The process is repeated until a desired level of classifier accuracy is achieved, or the budget available for feature acquisition is exhausted. 

They propose that specific solutions to the AFA problem differ based on the method used to score and rank queries. Scores are computed based on the *expected utility *of each query. The scoring function measures what the expected improvement is in the accuracy of a classifier if we know the value of a particular missing feature given the cost involved in obtaining it. Given that a feature value *f_i _*is missing for an instance and it can take any of the *K *values *(V_1_, V_2_, ... V_k_)*, its expected utility is measured as,

$$E\left(f_{i}\right)=\overset{K}{\underset{k=1}{\sum }}P\left(f_{i}=V_{k}\right)*U\left(f_{i}=V_{k}\right)$$

$$U\left(f_{i}\right)=\frac{A\left(F,f_{i}=V_{k}\right)-A\left(F\right)}{C\left(f_{i}\right)}$$

where *A(F, f_i _= V_k_) *is the accuracy of the classifier when it is trained with the value of *f_i _*set to *V_k_. A(F) *is the accuracy of the original classifier. *C(f_i_) *is the cost of acquiring the feature value. *P(f_i _= V_k_) *is measured by building a classifier *C_i _*corresponding to each feature. In the training data all the features other than *f_i _*and the class label are taken as feature values and *C_i _*is built. The classifier *C_i _*predicts what the probability is that a missing feature will take a particular value when the other feature values and the class label for an instance are known. It finds the expected utility for various missing values across all the instances. The missing feature with maximum expected utility is selected and its value is obtained (by experimentation or manual labeling, as applicable).

This method is computationally intensive for several classifiers types and for several domains. This is because the classifier needs to be trained for each missing feature and its various possible values in order to measure *A(F, f_i _= V_k_)*. Therefore, in order to evaluate the utility of a single missing feature of a given instance, the classifier is to be retrained '*K*' times. As this procedure is repeated for each of the missing feature elements, the classifier is to be retrained |M|*K times in a single iteration (where *M *is the set of all missing features over all instances). Although incremental learning can be done efficiently for classifiers like Naive Bayes, for several other classifiers it is inefficient. For instance in the case of Random Forests, retraining the classifier once has time-complexity of T*N*log(N) [17], where *T *is the number of trees in the random forest and *N *is the number of instances in the training data. So, the total time complexity for evaluating the utility of all the missing features is T*N*log N*|M|*k. When the dataset size is large and has several missing values, the time for evaluating the expected utility would be very high. To overcome this, the authors (Melville et al) proposed Sampled Expected Utility wherein a random subset of instances (*S*) with missing feature values are selected randomly and are evaluated by the above procedure. The results show that this expected utility approach performs better than the method which randomly picks missing feature values for labeling. Saar-Tsechansky et al. create the reduced consideration set '*S*' by giving preference to missing features in instances which are misclassified or instances which have high uncertainty as to their label according to the induced classifier model [18]. Though methods like sampled expected utility reduce the consideration set, for large data sets with several missing features this approach would be computationally very expensive, especially for models which are parametric. Gregory et al. proposed an active feature acquisition approach that they specifically evaluated on two sequence labeling tasks [19]. Their approach also required re-training of classifiers. Attenberg, Melville and Provost present a unified approach to active dual supervision, where they determine which feature or instance should be acquired that benefits the classifier the most by extending the sampled expected utility measures proposed for active dual supervision, but their methods still require re-training the classifiers [20].

In expected utility based approaches for AFA, the usefulness of acquiring a missing feature is estimated by retraining the classifier for each of the possible values that the missing feature can take and then calculating the expected improvement in classifier accuracy. However, retraining the classifier for every possible value, for each missing feature of each instance, is computationally very intensive, or even infeasible for large multi-dimensional data sets.

In this work we propose a novel heuristic to measure the utility of acquiring a missing feature value without the need of retraining of the classifier multiple times.

## Methods

### Proposed active feature selection strategy

Consider a training data set with N instances and a classifier '*C*' trained on this data. Say that a feature value *f_i _*is missing for a particular instance '*p*' in this training set and that it can take any of the *K *values (*V_1_,V_2_, ... V_k_*). Let (*L_1_, L_2_, ... L_N_*) be the various possible labels for the instance. We assume that the instance under consideration is already labeled to be *L_m_*. The expected utility of acquiring *f_i _*is measured as follows,

$$U\left(f_{i}\right)=\overset{K}{\underset{j=1}{\sum }}P\left(f_{i}=V_{j}|y=L_{m}\right)*\Delta \rho \left(f_{i}=V_{j}\right)$$

The estimated change *Δρ *is a heuristic to estimate how much of a change would be induced into the current classifier '*C*' if it is retrained with '*p*' having feature value *f_i _*set to *V_j_*. If the probability that '*p*' belongs to its correct class according to '*C*' decreases if *f_i _*takes the value *V_j_*, then it indicates that on retraining, the classifier '*C*' has to adjust its beliefs so as to increase the predicted probability of '*p*' belonging to its correct class (so as to reduce misclassification cost, or in classifiers like SVM to maximize the margin).

$$\Delta \rho \left(f_{i}=V_{j}\right)=P\left(y=L_{m}|C,p\right)-P\left(y=L_{m}|C,\left(p\cap f_{i}=V_{j}\right)\right)$$

*P(y=L_m _| C,p) *= predicted probability that '*p*' has label *L_m _*according to previously learnt classifier *C*.

*P(y= L_m _| C,(p ∩ f_i _= V_j_)) *= predicted probability that '*p*' has label *L_m _*according to previously learnt classifier *C*, when the feature *f_i _*of '*p*' is set to *V_j_*

If *Δρ *is less than 0, it indicates that when *f_i _*is set to *V_j _*it concurs with the belief of *C *(i.e. the estimated probability of '*p*' belonging to its correct class (*L_m_*) according to *C *increases). Hence in '*p*' if *f_i _*is set to *V_j _*and *C *is retrained, classifier is not expected to update its model. Therefore, *Δρ *is set to 0 for that case.

### Dataset and feature descriptors

In the domain of PPI prediction, there is no "negative dataset" available; that is, there are no pairs that are known to be non-interacting. However, in 500 to 1500 randomly selected pairs only one pair is expected to be an interacting pair [21]. Therefore, random pairs are usually treated as negative class instances in this domain. For our work, we created training and testing datasets of 10,000 protein pair instances each with 2,000 interacting pairs and 8,000 random pairs. AFA is carried out in batch mode, selecting 500 missing values in each batch.

### Gene ontology features

Given a protein pair, Gene Ontology (GO) information is usually encoded by measuring the semantic similarity between the GO terms of the proteins in the pair. But it is possible that a pair of GO terms (function, processes or cellular component) share low semantic similarity but they could be crucial for interaction between a protein pair. Hence we use existing protein interactions to estimate the value of a pair of GO terms for protein interaction. These estimates are then used to encode the new features. The protein interaction data was obtained from the HPRD data base [7] and the GO annotations from the GO database (http://www.geneontology.org). From this data, pairs of GO terms (GO1, GO2) and the number of protein interactions in which each pair occurs n(GO1,GO2) are computed. Let's say Protein A is associated with GO1, GO2 and Protein B is associated with GO4 and Protein A and Protein B interact. Then the frequency of the pairs (GO1, GO4), (GO2, GO4), are incremented. Then the feature value for a protein pair (P1,P2) is proportional to,

$$\underset{GO_{1}\in Set_{1}}{\sum }\underset{GO_{2}\in Set_{2}}{\sum }\frac{n\left(GO_{1}GO_{2}\right)}{n\left(GO_{1}\right)*n\left(GO_{2}\right)}$$

where, Set1 are the set of GO terms for P1 and Set2 are the terms for P2. Three feature values, one each by using GO annotations for biological process, cellular component and molecular function are developed.

### Gene expression

Gene Expression features for PPI prediction problem are usually generated from a limited set of gene expression experiments. Qi et al. use 16 gene expression experiments [11]. However, in our work we use the several thousand gene expression experiments available in the Stanford Microarray Database (SMD) to compute this feature [22]. Note that this feature needs to be computed for every possible protein pair (20,000 × 20,000/2 = 200 million pars, where 20,000 is roughly the number of proteins (genes) currently catalogued in the human protein reference database); the process therefore needs to be efficient. Several thousand experiments in the SMD have been divided into 70 categories. To prevent several thousand file reads for computing gene expression feature for a protein pair, we preprocess the gene expression data in each category into a single file. This file has for each protein a vector of gene expression values corresponding to the microarray experiment. So, 70 pre-processed files corresponding to the 70 categories are obtained. For a given protein pair (P1,P2), let GE1_m _be the vector of gene expression values corresponding to the category 'm' for protein P1 and let GE2_m _be the vector of gene expression values corresponding to the category 'm' for protein P2. Let N be the length of the vector. The Pearson Correlation Co-efficient is computed between these two vectors as follows,

$$\text{PP}{\text{C}}_{\text{m}}=\frac{\sum \text{GE}{\text{1}}_{\text{m}}*\text{GE}{\text{2}}_{\text{m}}-\left(\sum \text{GE}{\text{1}}_{\text{m}}*\sum \text{GE}{\text{2}}_{\text{m}}\right)/\text{N}}{\sqrt{\left(\sum \overset{\text{2}}{\underset{\text{m}}{\text{GE1}}}-\frac{{\left(\sum \text{GE}{\text{1}}_{\text{m}}\right)}^{\text{2}}}{\text{N}}\right)*\left(\sum \overset{\text{2}}{\underset{\text{m}}{\text{GE2}}}-\frac{{\left(\sum \text{GE}{\text{2}}_{\text{m}}\right)}^{\text{2}}}{\text{N}}\right)}}$$

Two gene expression features are computed. They are the mean and standard deviation of the correlation values (PPC_m_) for the 70 categories.

We can further improve the efficiency of the process by finding proteins which have little variance in correlations. Say if protein P1 does not have much variance, then for any protein P2 there will be little correlation between P1 and P2.

### Domain interaction feature

The domain interaction information is obtained from the InterDom database [23]. It has information about the list of domains belonging to each protein and an interaction score between pairs of domains. Given a protein pair (P1,P2), the domain interaction feature is calculated as follows,

$$\underset{d_{1}\in Set_{1}}{\sum }\underset{d_{2}\in Set_{2}}{\sum }\frac{score\left(d_{1}d_{2}\right)}{\left|D_{1}||D_{2}\right|}$$

where,

D1 is the set of domains in protein P1

D2 is the set of domains in protein P2

score(d1,d2) is the interaction score between the domains d1 and d2.

### Gene neighbourhood

For a given protein pair, this feature measures how close the genes (encoding the proteins) are to each other in the genome. The data for computing this feature is downloaded from ftp://ftp.ncbi.nlm.nih.gov/gene/. Based on the locus tag and the chromosome to which the genes are attached the distance score is computed between the genes.

### Tissue feature

The data for generating the tissue feature was obtained from Tissue-specific Gene Expression and Regulation (TiGER) database [24]. Information on proteins and the tissues in which they occur is retrieved. The tissue feature score for a protein pair (P1,P2) is computed as,

$$\frac{T_{1}\cap T_{2}}{\text{min}\left(T_{1},T_{2}\right)}$$

where,

T1 is the set of tissues P1 occurs in

T2 is the set of tissues P2 occurs in

Evaluation metric

The metrics that we employ here are those that are commonly used in the domain of information retrieval: F-score. F-score is the harmonic mean of the precision and recall. Precision is measured as the percentage of true positives among all predicted interactions; recall is the percentage of true positives among all real interactions.

## Results

### Experimental setup

The Gene Expression and Gene Neighborhood features in PPI prediction feature vectors have nearly 100% coverage, and therefore do not depend on active feature acquisition. The Gene Ontology features (biological process, cellular component and molecular function), domain and tissue features have a large number of missing values. So we considered these five features to study active feature acquisition for PPI prediction. We consider only protein pairs where individual proteins have gene ontology annotations and at least one of tissue or domain annotations. This is to ensure that the feature vector is reasonably filled. A training and test data set of 10,000 instances each was generated. The training set has 10,000 × 5 = 50,000 feature values of which nearly half of these feature values are missing in the original dataset. Additionally, we set another 10,000 feature values to be missing (which are otherwise available in the dataset), so as to simulate acquiring these features as-and-when asked by the algorithm. In other words, these are the feature values which are available for acquisition by the AFA system. To apply AFA, we need to discretize the real valued features. To do that we apply the commonly applied Maximum Description Length (MDL) based discretization method proposed by Fayyad and Irani [25]. We use the Weka Machine Learning Toolkit's implementation of this discretization method [26].

### Performance comparison

**Figure 1:**
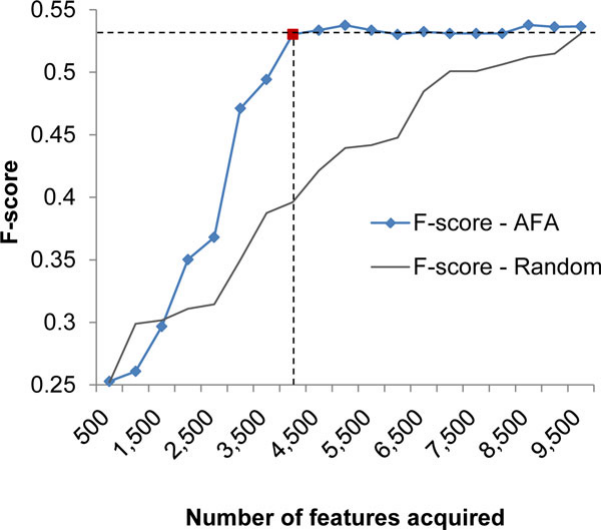
**F-score for Active Feature Acquisition**. X-axis shows the number of missing features acquired. Y-axis shows the F-score for the classifier built at the corresponding number of missing features acquired

We compared the performance of the proposed Active Feature Acquisition (AFA) heuristic with a system which randomly selects missing feature values for acquisition. In each iteration 500 missing values are acquired and a Decision Tree Classifier is retrained. The F-score of the classifiers generated by the 2 methods at the end of each iteration is compared (Figure 1). It can be seen that the AFA system achieves the peak F-score after acquiring about 4,200 missing feature values (indicated by red square marker on the figure). To achieve a similar F-score with a training data created with random-acquisition, almost 9,500 feature values had to be acquired. This shows that the AFA system is able to create an optimal training data much more economically, by asking for only 40% of the missing feature values.

**Figure 2:**
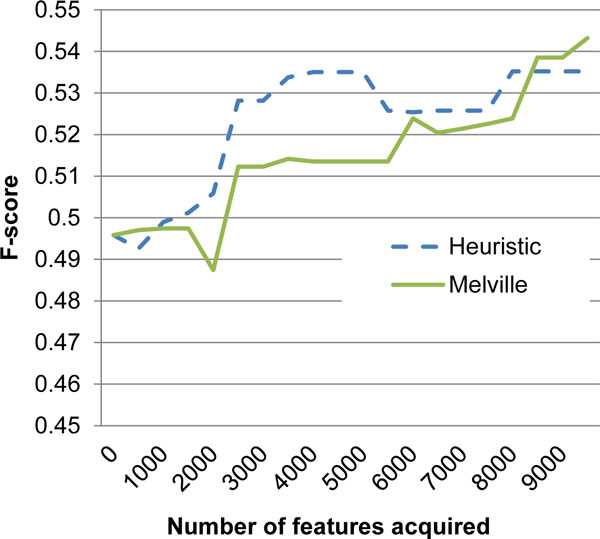
**Comparison with Melville et al's method**. The graph shows F-score of the heuristic method proposed here with that proposed by Melville et al. Axes descriptions are same as in Figure 1.

While the above comparison shows that the heuristic method is creating a training data effectively compared to random selection, it would be interesting to see whether this method performs comparably to computationally intensive AFA methods. To measure the relative performance, we compared the heuristic AFA method with that proposed by Melville et al., on the PPI dataset. Results are shown in Figure 2. We found that our method performs slightly better than the other method, and when combined with the fact that it does not require retraining the classifier numerous times, it clearly presents an advantage.

**Figure 3:**
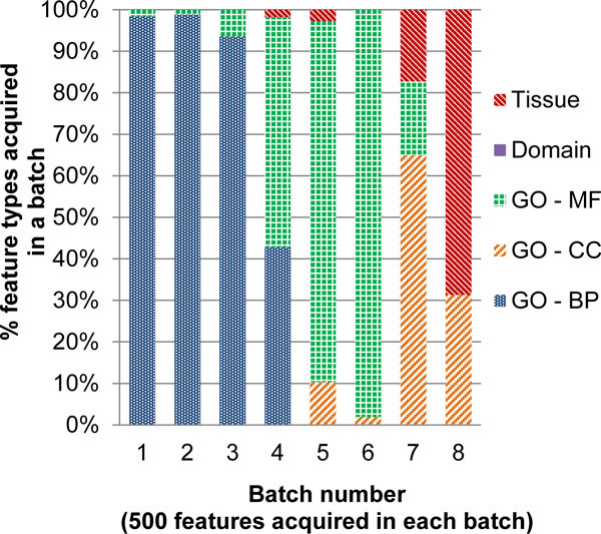
**Type of features acquired in different stages of active feature acquisition**. The X-axis shows the iteration number. The bars show the amount of missing values acquired for each feature type in that iteration.

Next, we analyzed what types of features are being selected for querying in the AFA procedure. Figure 3 shows how many missing values were acquired for each feature type in each batch. The results show that in the initial iterations missing values for biological process and molecular function features (which describe the functional similarity of the protein pair) are selected heavily and in the later iterations features related to localization (tissue and cell component features) are selected more. To understand this phenomenon, we studied the decision trees that were constructed in each iteration. The decision trees built have the Gene Ontology biological process and molecular function at the higher levels and the localization features in the lower levels of the tree. The functional similarity features are initially acquired in larger amounts till the top level rules are learnt and then missing localization features are obtained for further learning rules corresponding to lower levels of the tree.

### Performance of the proposed AFA heuristic method on other classification tasks

We carried out active feature acquisition on other standard classification tasks with data available at the UCI Machine Learning Repository (http://archive.ics.uci.edu/ml/datasets.html). However, the AFA method proposed here did not perform better in these cases (we have not tested Melville method, but tested AFA against random selection). It remains to be seen whether the proposed heuristic method has particular advantage in PPI-prediction like domains, i.e. when (i) the data has several missing values, or (ii) the positive instances are an extremely rare category among the unlabeled instances. Although this is discouraging, the proposed method presents a novel direction for estimating the utility, which is not dependent on training a classifier numerous times in each iteration. The evaluations on these datasets are as yet preliminary. Rigorous testing and analysis is to be carried out in future, with our method as well as previous methods, to understand what the domain-characteristics may be that lead to the success or failure by different methods in these domains.

## Conclusions

Active learning methods optimize the interaction between a computational method and a human expert by preselecting the data that an expert is to devote time or resources on, so that the outcome contributes most beneficially to the computational algorithm. Typically these methods are applied to domains that have massive amount of data such as astronomical images or world-wide-web documents, where, even though each data instance can be labelled with little manual effort, creation of a training data that is representative of the entire dataset can benefit with active learning approaches. In molecular biology domain however, the reasons for active learning are atypical. Here, even though the data may not be as massive, the resources, time and expertise required to characterize each instance is very large, making it impossible to characterize even moderately large datasets. For this reason, active learning methods can contribute to the domain of molecular biology, and guide the selection of molecule-experiment combinations that yield maximum benefit towards characterizing other molecules by computational methods. We have previously applied active learning for label acquisition for protein-protein interaction prediction [27].

Here, we presented a new heuristic approach for Active Feature Acquisition (AFA) that reduces computational cost by estimating the improvement a feature value would bring to the classifier. In contrast, other expected utility-based methods for feature acquisition train a new classifier for each 'instance-feature-value' triple. The results show that AFA achieves comparable F-score by acquiring only 40% as much missing features as the random method. Further, AFA has not been previously applied for PPI prediction (to the best of our knowledge) and the results show that AFA would be critical for the domain of PPI prediction where the biological features are missing for several protein pairs (especially for pairs with proteins which have not been studied extensively).

Active label/feature acquisition strategies generally work under budget constraints, and it is necessary to account for the cost of acquiring these missing values. The cost for experimentally determining the interaction of the protein pairs might vary for different pairs depending upon the localization of the proteins and the experimental conditions which need to be created to verify the interaction. Similarly cost of obtaining the missing features might differ for the various feature types. So it is necessary to develop computational methods which are able to model the cost of experimental annotation and incorporate them in to the active label/feature acquisition strategies [28].

The heuristic we proposed for active feature acquisition works in a batch mode selecting a group of missing features to be acquired in each iteration; further improvements can be achieved by incorporating marginal relevance of the features with respect to each other to ensure diversity in the selected missing features within a batch [29]. It would be interesting to see how to address active learning in domains with sparse-label and sparse-feature space. The Active Information Approaches proposed in [18] may be a starting point in this direction. The active learning and active feature acquisition approaches we considered evaluate the utility only at a particular instance/missing-feature level. It is possible that acquiring a particular pair of missing labels or features can bring in much higher utility than the sum of the utility of acquiring each of them individually. Further we may be constrained by the amount of budget we can spend to learn the classifier. However performing a complete look-ahead has exponential time complexity. So highly simplified look-ahead procedures such as single feature look-ahead (SFL) [30] and randomized single feature look-ahead (RSFL) [31] have been proposed. Developing advanced look-ahead policies that incorporate more information about the state space and deeper look-ahead would enable obtaining higher error reduction for the given budget.

## Competing interests

The authors declare that they have no competing interests.

## Authors' contributions

MT proposed the heuristic for calculating the utility of acquiring a missing feature and carried out the bulk of the experiments with direction and supervision from MG. TS compared the method with Melville et al. method. Manuscript has been prepared by MT and MG with relevant contribution from TS.
